# The Multiple Strategies of an Insect Herbivore to Overcome Plant Cyanogenic Glucoside Defence

**DOI:** 10.1371/journal.pone.0091337

**Published:** 2014-03-13

**Authors:** Stefan Pentzold, Mika Zagrobelny, Pernille Sølvhøj Roelsgaard, Birger Lindberg Møller, Søren Bak

**Affiliations:** Plant Biochemistry Laboratory and Villum research center ‘Plant Plasticity’, Department of Plant and Environmental Sciences, University of Copenhagen, Copenhagen, Denmark; Portland State University, United States of America

## Abstract

Cyanogenic glucosides (CNglcs) are widespread plant defence compounds that release toxic hydrogen cyanide by plant β-glucosidase activity after tissue damage. Specialised insect herbivores have evolved counter strategies and some sequester CNglcs, but the underlying mechanisms to keep CNglcs intact during feeding and digestion are unknown. We show that CNglc-sequestering *Zygaena filipendulae* larvae combine behavioural, morphological, physiological and biochemical strategies at different time points during feeding and digestion to avoid toxic hydrolysis of the CNglcs present in their *Lotus* food plant, i.e. cyanogenesis. We found that a high feeding rate limits the time for plant β-glucosidases to hydrolyse CNglcs. Larvae performed leaf-snipping, a minimal disruptive feeding mode that prevents mixing of plant β-glucosidases and CNglcs. Saliva extracts did not inhibit plant cyanogenesis. However, a highly alkaline midgut lumen inhibited the activity of ingested plant β-glucosidases significantly. Moreover, insect β-glucosidases from the saliva and gut tissue did not hydrolyse the CNglcs present in *Lotus*. The strategies disclosed may also be used by other insect species to overcome CNglc-based plant defence and to sequester these compounds intact.

## Introduction

Plants are often endowed with chemical defence compounds, of which some are permanently present in anticipation of an herbivore or pathogen attack. These constitutive plant defence compounds may be stored in a non-toxic glucosylated form and be spatially separated from their bioactivating β-glucosidases [1], [2]. This is known as a two-component defence system. The two components come into contact after tissue damage by herbivory or pathogenic fungi resulting in the immediate release of toxic aglucones [1], [2], [3]. Accordingly, two-component plant defence systems constitute a challenge to herbivores during feeding and digestion, but the innate conditional toxicity and permanent presence may be key factors for insect herbivores to evolve counter strategies, such as sequestration. Sequestration, the specific accumulation, storage and concentration of plant chemicals in the insect body [4], [5], is an efficient strategy, because glucosylated plant defence compounds become spatially separated from the plant β-glucosidases which are retained in the gut lumen [2].

Herbivorous insect species from several orders such as Lepidoptera (butterflies and moths), Coleoptera (beetles), Hemiptera (e.g. aphids) and Hymenoptera (e.g. sawflies) [4], [6] are known to sequester several classes of two-component plant chemical defence such as cyanogenic-, iridoid- and salicinoid glucosides as well as glucosinolates [6], [7], [8], [9], [10]. Yet it is unclear how insects keep these glucosylated plant defence compounds intact during feeding and passage through the digestive tract. It is hypothesized that the hydrolysis of the glucosylated compound in the gut must somehow be circumvented [11], for example by the exclusive presence of gut glucosidases that are inactive against these substrates [12]. Recent studies suggest that insects are able to interfere with either one or both components of the plant’s two-component chemical defence system [13], [14], [15], [16]. It is beneficial for the feeding insect to avoid plant β-glucosidase activity as this would keep the glucosylated defence compounds intact and avoid generation of toxic aglucones, and also retain the option to sequester. This requires several strategies at different time points during feeding and digestion [2]. No study has yet shown that such strategies occur in one single insect herbivore species, to our knowledge.

**Figure 1:**
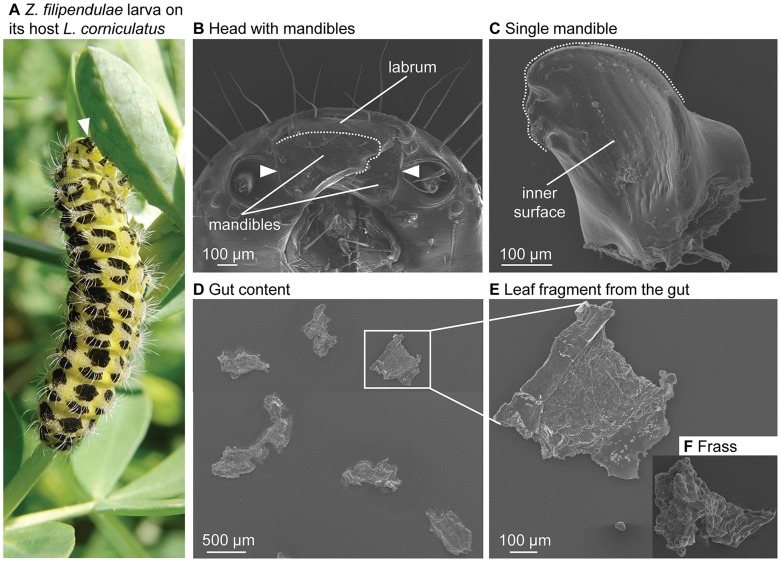
The mandible morphology of *Z. filipendulae* enables leaf-snipping to ingest and digest large leaf fragments. **A.** Larva of *Z. filipendulae* feeding on its host plant *L. corniculatus*, which contains the cyanogenic glucosides linamarin and lotaustralin. The mouthparts including the mandibles are indicated by an arrowhead. The larva is ∼ 2.5 cm long. **B.** Frontal-ventral view of the head with the two mandibles laying partly over each other. The distance between the bases of both mandibles is ∼ 600 µm (arrowheads). The leaf-processing area of the mandible is indicated by a dashed line. Both mandibles are partly covered by the labrum in a closed position. **C.** The right mandible viewed dorsally showing a round, concave and non-toothed shape with a length of ∼ 400 µm and a width of ∼ 300 µm. The leaf-processing area is indicated by a dashed line. **D.** The larval gut content shows that ingested *L. corniculatus* leaf fragments are relatively large and match the dimensions and morphology of the two mandibles. **E.** Detail of a representative *L. corniculatus* leaf fragment from the larval gut which is ∼ 550×450 µm - a similar size is retained in the frass (**F.**)**.**

**Figure 2:**
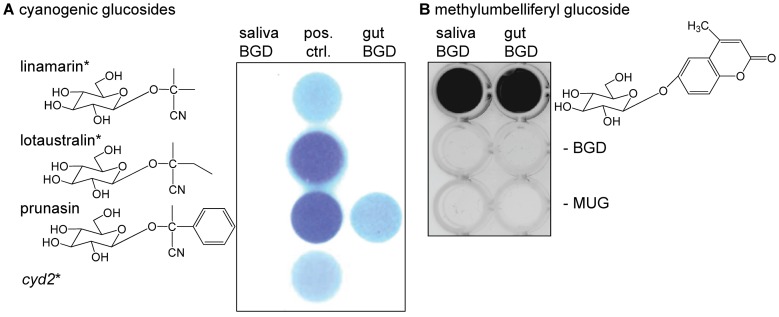
β-Glucosidases from saliva and gut tissue of *Z. filipendulae* do not hydrolyse linamarin and lotaustralin. **A.** Hydrolysis of cyanogenic glucosides (CNglcs) with corresponding HCN release is visualized by Feigl-Anger paper, or **B.** via fluorescence of methylumbelliferone, the hydrolysis product of the generic substrate 4-methylumbelliferyl β-D-glucopyranoside (MUG; in black). The β-glucosidases (BGDs) extracted from the saliva and gut are active enzymes as they hydrolyse MUG (**B.**), as well as prunasin in case of the gut β-glucosidase (**A.**). Importantly, the two CNglcs linamarin and lotaustralin present in the food plant *L. corniculatus* (indicated by *) are not hydrolysed by the saliva and gut β-glucosidases. Linamarin and lotaustralin are neither hydrolysed if tested individually (**A.**, top), nor hydrolysed if tested using a *cyd2* leaf macerate (**A.**, bottom). A macerate of *L. japonicus cyd2* mimics digestion of a leaf containing linamarin and lotaustralin, but does not release HCN as it lacks the corresponding BGD.

Cyanogenic glucosides (CNglcs) are a widespread class of two-component plant chemical defence. In intact plant tissue, CNglcs and their corresponding β-glucosidases are spatially separated. After tissue damage by herbivory both components mix and quickly release toxic hydrogen cyanide (HCN), i.e. cyanogenesis [17], [18], [19]. It is only known in a few cases how insect herbivores overcome plants defended by CNglcs. The Neotropical Sara longwing (*Heliconius sara*) metabolizes the CNglc epivolkenin from its food plant by replacing the nitrile group with a thiol group, which prevents HCN release [20]. An alternative strategy to overcome toxicity of plant CNglcs would be to avoid mixing of the CNglcs with the corresponding plant β-glucosidases. Larvae of the specialised six-spot burnet moth *Zygaena filipendulae* (Lepidoptera: Zygaenidae) feed on *Lotus* spp. (mainly *Lotus corniculatus*, Fabaceae) plants defended by the CNglcs linamarin and lotaustralin (Fig. 1A, Fig. 2A), and sequester these compounds in an intact glucosylated form [10]. For optimal growth and development larvae are heavily dependent on sequestration of linamarin and lotaustralin [21], and if they cannot sequester enough, they will start to *de novo* biosynthesise them [22], [23]. Larvae constantly emit HCN by metabolism of linamarin and lotaustralin as part of their defence against predators [21], [24]. However, it is unknown how hydrolysis of CNglcs is avoided during feeding and digestion, which is a prerequisite for sequestration.

Here we provide evidence that *Z. filipendulae* larvae have evolved multiple strategies that are used at different time points during feeding and digestion to overcome toxicity of plant CNglcs. Most of these strategies target plant β-glucosidase activity, which ensures that the larvae can sequester CNglcs intact. The strategies disclosed are likely to constitute key principles also employed by other lepidopteran species and insect herbivores from different orders that feed on plants containing other classes of two-component chemical defence.

## Results

### A High Feeding Rate and a Leaf-snipping Feeding Mode Strongly Limit CNglc Hydrolysis

We measured the feeding rate of *Z. filipendulae* and found that larvae consume 3.8 cm^2^ (±0.2 SE) of *L. corniculatus* leaves per hour. As the extent of CNglc hydrolysis during feeding is also dependent on the shape of the mandibles [2], we dissected and analysed mouthpart and mandible morphology via scanning electron microscopy (Fig. 1). We found that mandibles are simple, round and mainly non-toothed in shape. The distance between the base of both mandibles was ∼ 600 µm (Fig. 1B), and each mandible had a length of ∼ 400 µm and a width of ∼ 300 µm (Fig. 1C). The morphology of the mandibles enabled larvae to snip, ingest and digest leaf fragments from *L. corniculatus* with dimensions up to 550 µm×450 µm (0.25 mm^2^) (Fig. 1D, E). Leaf fragments of similar sizes were observed in the frass (Fig. 1F).

Because *Z. filipendulae* larvae eat at a high rate and only cause minimal damage to the plant tissue during feeding, it was of interest to directly quantify CNglc hydrolysis that occurs during feeding (Table 1). To test this, we first determined the background release of HCN from larvae using the *Lotus japonicus* mutant *cyd2*. This mutant is in the *L. japonicus* wild-type (MG-20) background and thus contains linamarin and lotaustralin, but does not release HCN as it lacks the corresponding β-glucosidase [25]. HCN emission from intact *cyd2* leaves was insignificant and from crushed *cyd2* leaves 1.17 nmol (±0.73 SD) HCN was released on average. However, when larvae ate on *cyd2* leaves 1.50 nmol (±1.03 SD) HCN was released. Consequently, 0.33 nmol (±1.26 SD) must be derived from the HCN emission of the larva themselves (Table 1). When subtracting this amount from the total HCN emission of larvae feeding on MG-20 leaves (1.62 nmol ±0.97 SD), HCN emission from MG-20 damaged by feeding can be estimated to 1.29 nmol (±1.59 SD). The total HCN potential per ingested MG-20 leaf was 119.4 nmol (±47.1 SD), i.e. 1.1% (±1.0 SD) of the total leaf CNglcs was hydrolysed in the course of feeding.

**Table 1:** CNglc hydrolysis in *Lotus* leaves is minimal during larval feeding.

HCN (nmol)	intact	crushed	feeding by *Z. filipendulae*
***cyd2***	0.02±0.04	1.17±0.73	1.50±1.03
**MG-20**	0.09±0.12	119.4±47.10	1.62±0.97 (1.29±1.59*)

HCN emission as measurement of CNglc hydrolysis from *L. japonicus cyd2* and MG-20 leaves with no damage (intact), with mechanical damage (crushed) or with damage by feeding *Z. filipendulae* larvae. Values are given as mean with ± SD, N = 11. (*After subtraction of HCN emission from larvae [ = 0.33 nmol ±1.26 SD], which is the mean value of HCN emission ± SD from feeding on *cyd2* minus the mean value of HCN emission ± SD from crushed *cyd2*).

### A Highly Alkaline Midgut Lumen Inhibits Plant β-glucosidase Activity and Prevents CNglc hydrolysis

**Figure 3:**
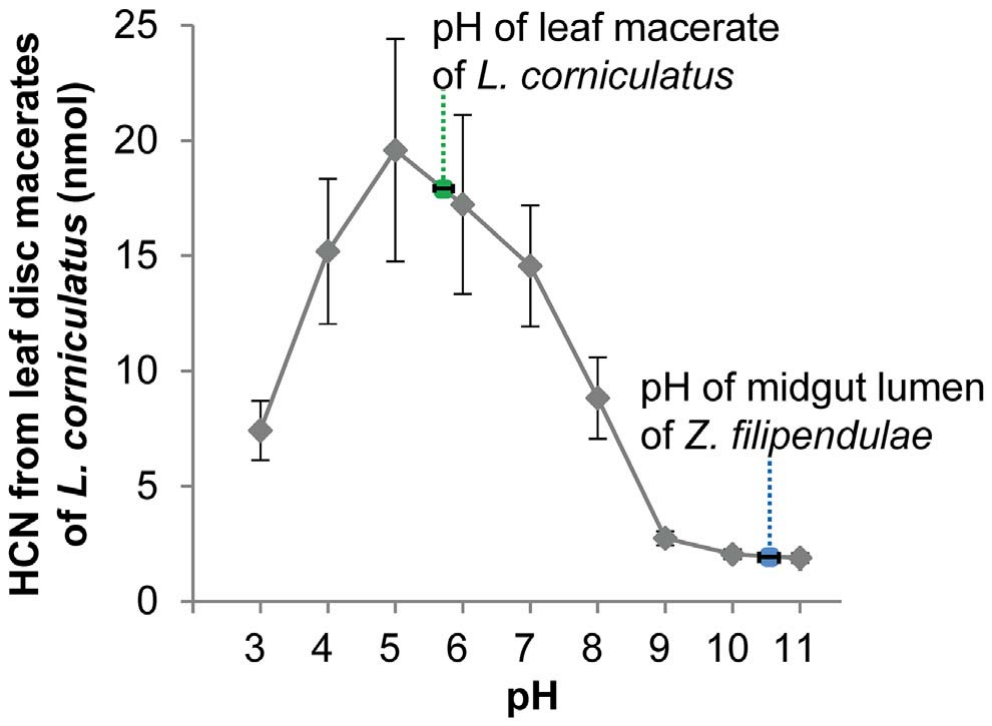
HCN emission from *L. corniculatus* leaf macerates is strongly reduced in the highly alkaline midgut. The pH of *L. corniculatus* leaf macerates is slightly acidic (5.9±0.1 SD, N = 10, green dotted line), whereas the pH measured in the midgut lumen of *Z. filipendulae* larvae is highly alkaline (10.6±0.1 SD, N = 11, blue dotted line). HCN emission from leaf disc macerates is highest at pH 5–6, which matches the pH of *L. corniculatus* leaf macerates. However, HCN emission is significantly reduced under highly alkaline conditions at pH 10–11 present in the midgut lumen of *Z. filipendulae* larvae (one-tailed Student’s t-test, P<0.001). Each data point represents the mean (±SE) of ten independent incubations, i.e. 90 leaf discs were analysed in total.

During digestion and disruption of leaf material in the midgut, CNglcs and plant β-glucosidases may come into contact with each other in the midgut lumen. Mixing of both components could potentially result in hydrolysis of the CNglcs and generation of toxic HCN. However, we found that CNglc hydrolysis and HCN emission was strongly inhibited at the highly alkaline pH of 10.6 (±0.1 SD) measured in the midgut lumen of the larvae, in comparison to HCN emission at the pH of 5.9 (±0.1 SD) measured in *L. corniculatus* leaf macerates (Fig. 3). HCN release from leaf macerates, mediated by plant β-glucosidase activity, was efficient under slight acidic conditions at pH 5 (19.6 nmol ±4.8 SE) or 6 (17.2 nmol ±3.9 SE), but strongly reduced under high alkaline conditions at pH 10 (2.0 nmol ±0.2 SE) or 11 (1.9 nmol ±0.2 SE). These differences were highly significant (P<0.001, one-tailed Student’s t-test).

### Insect β-glucosidases from the Saliva and Gut Tissue Lack Activity Towards Plant CNglcs

Insects may possess endogenous β-glucosidases, which often function as digestive enzymes [26], [27]. To test whether the *Z. filipendulae* larvae produce β-glucosidases able to hydrolyse CNglcs, protein extractions from the salivary glands and gut tissue of *Z. filipendulae* larvae were prepared. The β-glucosidases from the saliva and gut of *Z. filipendulae* larvae hydrolysed 4-methylumbelliferyl β-D-glucopyranoside, a generic glucoside substrate used to monitor β-glucosidase activity (Fig. 2B). The gut β-glucosidase hydrolysed also prunasin, a CNglc found in almonds *Prunus* spp. (Fig. 2A). Importantly, the β-glucosidases from the saliva and gut did not hydrolyse linamarin and lotaustralin (Fig. 2A), the two CNglcs present in the larval food plant of *Z. filipendulae*.

**Figure 4:**
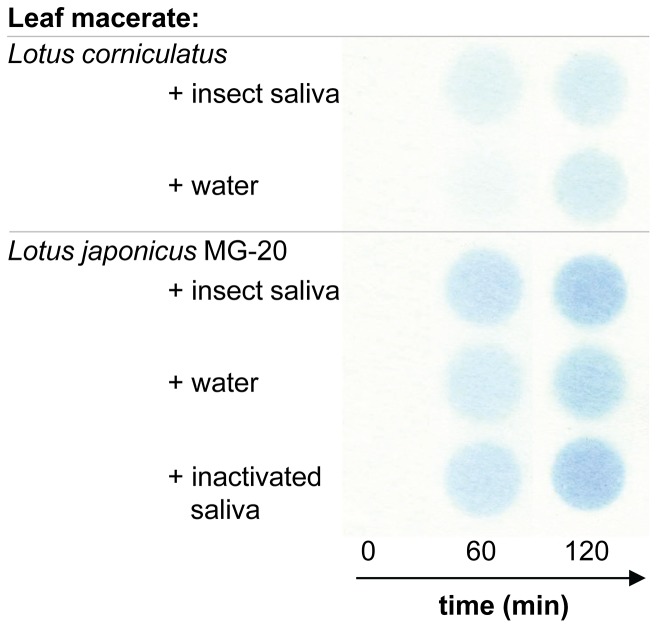
Saliva extracts of *Z. filipendulae* do not inhibit plant cyanogenesis. Feigl-Anger paper showing HCN emission over time from leaf macerates of *L. corniculatus* and *L. japonicus* (wild-type MG-20) incubated with either: insect saliva of *Z. filipendulae* larvae, water or heat-inactivated saliva as control (latter only on MG-20). When leaf macerates of both *Lotus* species are mixed with insect saliva, HCN emission increases at a similar rate as the leaf macerate incubated with water or heat-inactivated saliva.

Saliva is the first digestive substance that comes into contact with plant material. We tested if there are any substances present in the saliva that may inhibit CNglc hydrolysis. Therefore, leaf macerates from *L. corniculatus* and *L. japonicus* (wild-type MG-20) were incubated with saliva of *Z. filipendulae* larvae (Fig. 4). HCN emission increased over time in similar rates as seen from the water control and heat-inactivated saliva demonstrating that there are no apparent inhibitory constituents for plant cyanogenesis present in the larval saliva.

## Discussion

Cyanogenesis in plants mainly depends on the amount of tissue damage caused by an herbivore and on the time available for the β-glucosidase to hydrolyse CNglcs [1], [2]. Thus, the way insect herbivores process cyanogenic leaves is expected to impact on the effectiveness of CNglc-based plant defence.

We found that larvae of *Z. filipendulae* feed at higher rates (3.8 cm^2/^h ±0.2 SE) than reported for other lepidopteran species feeding on other plant species than *L. corniculatus*. For example, specialised *Manduca sexta* caterpillars feeding on tomato (*Solanum lycopersicum*) leaves do so at less than half of the rate of *Z. filipendulae* [28], although *M. sexta* is approximately twice the size as *Z. filipendulae*. Generalist lepidopterans, with approximately the same size as *Z. filipendulae* such as *Spodoptera exigua* or *Helicoverpa zea*, eat only around 2.2 cm^2^ (±0.3 SE) of acyanogenic corn leaves (*Zea mays*) per hour or eat other plants even slower such as CNglc-containing *Phaseolus vulgaris* (0.8 cm^2^/h ±0.4 SE) [28], [29]. Thus, we suggest that the comparatively high feeding rate of *Z. filipendulae* larvae significantly reduces the time period available for the plant β-glucosidase to hydrolyse linamarin and lotaustralin during the feeding phase.

The extent to which CNglcs may be hydrolysed in the course of feeding and ingestion of the plant material is also dependent on the morphology of the mandibles [2], as it determines the size and shape of the ingested leaf fragments. We found that the dimensions of the ingested leaf fragments are relatively large and match the dimensions and morphology of the two mandibles (Fig. 1). This shows that *Z. filipendulae* larvae snip leaves rather than chewing them [30], [31]. This so-called leaf-snipping is minimal disruptive, keeps most of the ingested plant cells intact, limits plant tissue damage and consequently prevents mixing of CNglcs and β-glucosidases [2], [31]. This keeps CNglcs from *L. corniculatus* intact during feeding and digestion by *Z. filipendulae*. The mandible morphology of other less specialised species belonging to Zygaenoidea (*Aglaope infausta* or *Heterogynis penella* which feed on cyanogenic and non-cyanogenic plant species) differs as their mandibles are more toothed and compact [32], [33], [34], [35]. In general, leaf-snipping lepidopterans have simple, round-shaped and non-toothed mandibles, which enable them to ingest plant fragments of a similar size [30], [31] as observed in *Z. filipendulae*.

To show that a high feeding rate and leaf-snipping result in limited CNglc hydrolysis during feeding, the degree of CNglc hydrolysis that occurs during feeding of *Z. filipendulae* larvae on *Lotus* plants was quantified (Table 1). This demonstrated that as little as 1.1% (±1.0 SD) of the total leaf CNglcs are hydrolysed in the course of feeding. This percentage is lower than found in other insect herbivore species feeding on cyanogenic plant material. For example, feeding of the lepidopteran ugly nest caterpillar (*Archips cerasivoranus*) and the fall webworm (*Hyphantria cunea*) on cherry (*Prunus*) species, results in emission of more than 2.5% and 10% of the total leaf HCN potential, respectively [36], [37]. Feeding of the orthopteran desert locust (*Schistocerca gregaria*) on lima beans (*Phaseolus lunatus*) results in emission between ∼ 2.5% and 15% of the HCN present in consumed leaf material, depending of the cyanogenic capacity and potential of the plant cultivar [38]. In contrast, HCN emission caused by feeding of desert locusts is considerably lower than caused by feeding of Mexican bean beetles (*Epilachna varivestis*) on the same plant species [38]. These differences can be linked to their different feeding modes: whereas desert locusts are leaf-snipping, bean beetles are leaf-chewing and cause more tissue damage [38]. These studies support the notion that processing of leaves by a leaf-snipping feeding mode, and at a high feeding rate, efficiently prevents CNglc hydrolysis.

As the foregut of lepidopteran larvae is only rudimentary, *Lotus* leaf fragments are quickly transported into the midgut for subsequent digestion. The midgut is the largest and most permeable part of the digestive tract, the main site of nutrient absorption and sequestration, but also target site for natural toxins and most insecticides [4], [27], [39]. Physiological conditions in the midgut, such as an alkaline pH, would thus be expected to dramatically influence the fate of the ingested CNglcs.

In the midgut, insect digestive enzymes such as lipases gain access to uncrushed leaf fragments and cell constituents by simple diffusion favoured by the dynamic movements of the lumen content. The enzymes disrupt the membranes and lipid bodies in leaf fragments, and as a result, nutrients in the form of proteins, soluble carbohydrates and metabolites diffuse out of the plant cells in a form available for absorption by the insect [31], [40]. CNglcs would leak out from the vacuole and other vesicles together with the cyanogenic β-glucosidases mainly localized in the apoplast. Both components would come into contact with each other in the midgut lumen and potentially result in hydrolysis of the CNglcs and generation of toxic HCN [1]. However, we find that plant β-glucosidase activity and thus CNglc hydrolysis and HCN emission are significantly reduced at the highly alkaline pH present in the larval midgut lumen (Fig. 3). Feeding herbivores which are not able to inhibit plant β-glucosidase activity would be exposed to high HCN emission. Thus, the highly alkaline midgut lumen keeps the CNglcs linamarin and lotaustralin largely intact during digestion, which prevents toxic HCN release and provides the basis for the larvae to sequester intact CNglcs. In agreement with this, digested plant material of *L. corniculatus* that has been in the gut of *Z. filipendulae* even for several hours still contains high amounts of intact linamarin and lotaustralin [16]. The minor amounts of HCN released in the midgut lumen would be detoxified via a β-cyanoalanine synthase [16], [41]. β-Glucosidases often have a tightly folded core structure, which enables activity over a wide range of pH and resistance to degradation for example by ionic detergents or proteases [42]. This general high stability of β-glucosidases could explain why even highly alkaline conditions in the midgut lumen of *Z. filipendulae* may not fully inhibit plant β-glucosidase activity resulting in minor hydrolysis of linamarin and lotaustralin (Fig. 3).

A similar inhibition of plant cyanogenesis by a highly alkaline midgut pH has only been reported in a few cases such as the ugly nest caterpillar or the fall webworm larva feeding on cherry [36], [37]. Highly alkaline conditions in the insect midgut may also inhibit plant β-glucosidases known from other two-component plant defence systems. For example, larvae of the fall armyworm (*Spodoptera frugiperda*) are able to feed on corn leaves which mainly contain the benzoxazinoid glucoside DIMBOA-glucoside. A midgut lumen of pH 10 was shown to decrease the release of toxic DIMBOA by more than 80% [40]. Caterpillars of the generalist winter moth (*Operophtera brumata*) succesfully feed on willow species that produce the salicinoid glucoside salicortin as a defence compound. During digestion in the alkaline midgut lumen of pH 9.5, salicortin is converted into a less complex glucoside, salicin [43], [44]. However, at this pH, salicin hydrolysis by β-glucosidases into its toxic aglucone is markedly reduced, as these enzymes have pH optima around pH 5 [45]. Thus, the alkaline midgut inhibits *Salix* β-glucosidase activity, which reduces release of toxic aglucones and enables larvae to ingest salicortin and to excrete non-toxic salicin in the frass [44].

A highly alkaline midgut lumen is known from numerous larvae of lepidopteran species [27], [46], [47], many of whom feed on plants not protected by two-component plant chemical defences [43]. Thus, a highly alkaline midgut was probably not an evolutionary response to two-component plant chemical defences, but rather herbivores with an alkaline midgut were pre-adapted to feed on plants protected by two-component chemical defences [2]. This might in turn have facilitated evolution of mechanisms for sequestration, including expression of required glucoside transporters [48]. The highly alkaline gut conditions furthermore allow insects to release hemicelluloses efficiently from plant cell walls [27], [49], and to optimally solubilize leaf proteins and cell wall polysaccharides during digestion [50]. Consequently, insect digestive enzymes such as proteases, amylases and lipases are well adapted as they often have alkaline pH optima [51], [52], [53], [54].

Insects often possess endogenous β-glucosidases, which function mainly as digestive enzymes [26], [27]. In the digestive tract, β-glucosidases from lepidopteran species are often trapped in the glycocalyx lining the midgut cells [55]. Thus, they are bound to the epithelial tissue, where more neutral pH values allow efficient hydrolytic activity [40], [47], [56], [57], irrespective of the highly alkaline gut lumen matrix where they were extracted [55]. Presence of promiscuous β-glucosidase activity might be anticipated to hydrolyse plant β-glucosides including the CNglcs [2]. Lack of insect β-glucosidases able to hydrolyse CNglcs would keep the CNglcs intact and avoid HCN release during midgut passage of the ingested CNglc-containing plant material. Our finding that the β-glucosidases from the salivary glands and gut tissue did not hydrolyse linamarin and lotaustralin (Fig. 2A), indicates that the catabolic system of the *Z. filipendulae* larvae is able to discriminate between the beneficial ability to hydrolyse nutritive plant glucosides and hydrolysis of linamarin and lotaustralin. β-Glucosidases from the saliva and gut of *Zygaena trifolii* larvae have previously been reported to lack the ability to efficiently hydrolyse CNglcs, whereas β-glucosidases in their haemolymph are highly active towards CNglcs [24]. A similar lack or reduction of β-glucosidase activity towards dietary CNglcs is reported from a few other lepidopterans such as *S. frugiperda* or the sugar cane borer *Diatraea saccharalis*, which enables these generalists to survive on an artificial diet containing the CNglc amygdalin [58], [59]. At the same time hydrolytic activities of the β-glucosidase towards plant oligosaccharides or cellulose are maintained [58], [59].

Saliva constitutes the first digestive substance that comes into contact with plant material. Insect herbivores may possess salivary inhibitors such as glucose oxidase to prevent production of plant chemical defence such as nicotine, probably by inhibiting the wound-signalling compound jasmonic acid [60], [61]. However, in the *Z. filipendulae* saliva we did not detect constituents that inhibit plant cyanogenesis (Fig. 4). It does not seem beneficial for *Z. filipendulae* larvae to produce salivary inhibitors for cyanogenesis, probably because salivary components and enzymes often play only a minor role in digestion in comparison to digestive enzymes from the midgut [27], [55], and because plant CNglcs pre-exist in anticipation of an insect attack. In *Z. filipendulae* larvae, plant material is ingested in relatively large fragments due to their leaf-snipping feeding mode, and quickly transported into the midgut [27], [39] where the highly alkaline pH acts as an efficient inhibitor for cyanogenesis.

## Conclusions

A key strategy for insect herbivores to overcome plant CNglc defence is to avoid mixing of CNglcs and their corresponding β-glucosidases, mainly by keeping plant cells and tissue intact during feeding. This is facilitated by *Z. filipendulae* larvae by combining a high feeding rate with a leaf-snipping feeding mode. An important factor during digestion of plant material is the inhibition of plant β-glucosidases, key enzymes in CNglc defence [1]. Plant β-glucosidases are often the main target for adapted insect herbivores [2], and are in case of *Z. filipendulae* larvae kept largely inactive by a highly alkaline midgut lumen. A further strategy is to avoid activity of insect β-glucosidases from different tissues towards plant CNglcs. These multiple strategies enable *Z. filipendulae* larvae to overcome the conditional toxicity of plant CNglcs and to sequester these compounds intact. Our study furthermore encourages that several research questions involving predation and herbivory of cyanogenic plants need to be examined in more detail.

The strategies disclosed could also be used by other lepidopterans and potentially by herbivorous insect species from different orders to overcome other classes of two-component plant defences activated by β-glucosidases. Avoiding mixing of both components and inhibiting plant β-glucosidase activity during feeding, ingestion and digestion would prevent generation of detrimental and toxic aglucones. This would enable insects to sequester these compounds in an intact and glucosylated form.

## Materials and Methods

### Ethics Statement

No specific permissions were required for collecting *Z. filipendulae* larvae or *L. corniculatus* plants in the south-west of Taastrup (55.65° N, 12.30°E), greater Copenhagen area, Denmark as both species are not endangered. Authors maintained the population at sustainable levels.

### a) Insect and Plant Material

Larvae of *Z. filipendulae* and *L. corniculatus* plants were collected from a natural population in the south-west of Taastrup (55.65° N, 12.30°E), greater Copenhagen area, Denmark in June 2011, 2012 and 2013. In the laboratory, larvae were kept in plastic boxes at room temperature and supplied with *L. corniculatus* food plants *ad libitum*. *L. corniculatus* plants have a ratio of ∼ 70∶30 of the CNglcs linamarin:lotaustralin and were grown in a greenhouse at 22°C. *Lotus japonicus* wild-type (accession MG-20) and the mutant line *cyd2* were germinated from seeds on filter paper and grown in soil under a 16 h light cycle. The *cyd2* mutant is in the MG-20 genetic background [25], and importantly for this study, both MG-20 and *cyd2* contain similar ratios (∼1∶34) and amounts of linamarin and lotaustralin [62]. *Cyd2* lacks the corresponding β-glucosidase designated LjBGD2 to hydrolyse CNglcs, and thus does not release HCN after tissue damage [24].

### b) Feeding Rate

*L. corniculatus* leaflet area was determined using digital high resolution photos taken from fresh leaflets and using ImageJ version 1.45 (http://rsbweb.nih.gov/ij/). Larvae of *Z. filipendulae* (N = 25, average weight 386±94 mg, stage L6) were starved for 2 h, presented with a leaflet, and the time to consume one leaflet was determined to calculate the average feeding rate including standard error in cm^2^/h.

### c) Morphological Analysis of Head Capsule, Mandibles, Gut Contents and Frass

Larvae were ice-chilled, anesthetized with CO_2_ and dissected under an EZ4 (Leica) stereo microscope using an E3340 SCS stitch fine mini of E3343 (Storz Instruments) and micro forceps BD330R (0.2 mm/110 mm, Braun, Aesculap). The following tissues were obtained: head capsule, mandibles and gut from which the content had been dissected. Frass was taken directly from defecating individuals. Plant material from the gut and frass as well as larval head capsule and mandibles were plated out and air-dried in a petri dish. Samples were then mounted on aluminium stubs using carbon tabs and sputter coated with a 1∶1 gold-palladium mixture. The specimens were observed in a Quanta 200 SEM scanning electron microscope (FEI Company) at 10–15 kV.

### d) CNglc Hydrolysis and HCN Emission from Plants During Feeding

To determine larval HCN emission, single L6 larvae (N = 24) were placed in sealed plastic boxes (8×6×5 cm) for 18 h each with one fresh *L. japonicus cyd2* leaf (contains CNglcs but lacks the corresponding β-glucosidase and thus does not emit HCN). As a negative control, HCN emission was also monitored from single fresh, intact *cyd2* leaves (N = 24) of the same size positioned in sealed boxes for 18 h without larvae. Afterwards, the same leaves were analysed for HCN emission by crushing them in 60 mM citric acid buffer (pH 6) in a 2 ml safe-lock tube using TissueLyser II (Qiagen) and leaving the tube open in a sealed plastic box for 18 h. To determine the HCN emission from plants during feeding, the same procedure with the same larvae (N = 24, fed for 1 d in between on *L. corniculatus* to re-adapt) was conducted using CNglc containing *L. japonicus* MG-20 plants (N = 24) which have a corresponding β-glucosidase and thus emit HCN after tissue damage. In the two experimental series, larval feeding intensity was monitored and only those experiments, in which the same larvae fully consumed the leaves were taken into account (2×N = 11). In all approaches, emitted HCN gas was trapped in 240 µl 1 M NaOH in a PCR tube mounted in the plastic box and quantified based on the colorimetric method described by [63] as modified by [64] using a SpectraMax M5 Microplate reader (Molecular Devices). Mean values with standard deviation were calculated.

### e) pH Measurements of Midgut Lumen and Leaf Macerate and CNglc Hydrolysis at Different pH Values

Dissection of the intact and full midgut of *Z. filipendulae* L6 larvae was carried out as described in materials and methods *c*). The luminal pH of eleven larvae was measured with a 100 µm-diameter Beetrode NMPH1 pH electrode and a 450 µm-diameter Dri-Ref 450 reference electrode (World Precision Instruments) on the intact anterior, middle and posterior midgut, and mean values with standard deviation were calculated. A Bee-Cal compensator (World Precision Instruments) was used to measure pH on a Hanna 211 pH meter (Hanna Instruments). For pH measurement of *L. corniculatus* leaf macerates (N = 10), 400 mg leaf tissue was ground in liquid nitrogen, diluted with 3 ml double distilled water, and pH measured on a Hanna 211 pH meter with a glass-body combination pH electrode HI 1131B (Hanna Instruments). To measure HCN emission from *L. corniculatus* leaf macerates at different pH values, two *L. corniculatus* leaf discs with a diameter of 6 mm from one plant were crushed by homogenization in a 2 ml safe-lock tube using TissueLyser II (Qiagen) in 500 µl buffer ranging from pH 3 to pH 11 [40 mM citric acid (pH 3), 55 mM citric acid (pH 4), 100 mM citric acid (pH 5), 60 mM citric acid (pH 6), 65 mM phosphate (pH 7), 12.5 mM borax (pH 8–10), 100 mM boric acid (pH 11)]. Leaf discs from ten different plants were measured at each different pH value. After 3 min centrifugation at 20.000 g, samples were incubated for 1 h at 30°C. HCN was quantified based on the colorimetric method described by [63] as modified by [64] using a SpectraMax M5 Microplate reader (Molecular Devices). Mean values with standard error were calculated; HCN emission at different pH values was tested for significant differences using one-tailed Student’s t-test (SigmaPlot 12).

### f) Salivary Glands and Gut Tissue: Extraction and β-glucosidase Activity

Salivary glands and gut tissue from two L6 larvae were dissected as described in *c*). After the gut was cut open, both tissues were thoroughly rinsed with ice-cold double distilled water and weighed. For extraction of β-glucosidases, the tissue was homogenized in ice-cold insect saline solution [25 mM NaCl, 5 mM KCl, 2 mM CaCl, 2,5 mM NaHCO_3_ (pH 7)] by grinding in 30 µl/mg tissue with an ice-cold pestle, ice-cold mortar and acid-washed sand. For efficient extraction of β-glucosidases, which are mostly attached to the glycocalyx [55], [65], samples were frozen and thawed three times before centrifugation for 45 min at 10.000 g at 4°C. Resulting supernatants were collected and used as a β-glucosidase-source. Protein concentration of the supernatant was determined by measuring absorbance at 280 nm on a NanoDrop ND-1000 (Thermo Scientific). To assay for cyanogenic β-glucosidase activity, saliva and gut homogenates (each 1.5 and 30 µg) were incubated in 150 µl of 65 mM phosphate buffer (pH 7) containing 500 µM of the CNglcs linamarin (Sigma-Aldrich 68264), lotaustralin (synthesized in our lab and kindly provided by M.S. Motawia) or prunasin (Sigma-Aldrich SMB00173). Homogenates were also incubated with a macerate of *cyd2*. Therefore, 40 mg leaves were crushed in 450 µl 65 mM phosphate buffer (pH 7), centrifuged and the supernatant was used. Incubation mixtures were added to a 96-well plate, fitted with a Feigl-Anger paper on top, sealed and incubated at room temperature for up to 18 h. The Feigl-Anger paper turns blue when exposed to hydrogen cyanide [25], [66]. Two types of control experiments were performed. As positive control for hydrolysis of linamarin, lotaustralin and the *cyd2* macerate, an extract containing the β-glucosidase LjBGD2 was used (from transient expression of the *LjBGD2* cDNA in *Nicotiana benthamiana*, see [25]), and for prunasin 0.1 U of β-glucosidase extracted from almonds (*Prunus dulcis*) (Sigma-Aldrich G-8625) was used. As negative control, the same incubations were carried out, but without any enzymes added. Feigl-Anger paper was prepared by wetting Whatman 3MM paper (GE Healthcare) in a 5 g per l chloroform solution of copper ethylacetoacetate (Alfa Aesar) and 4,4′-methylenebis(N,N-dimethylaniline) (Sigma-Aldrich M44451). The paper was wrapped in aluminium foil and stored at 4°C until use. To test for general β-glucosidase activity, saliva and gut extractions were incubated in 100 µl 65 mM phosphate buffer (pH 7) containing 500 µM 4-methylumbelliferyl β-D-glucopyranoside (MUG, Sigma M 3633) for 1 h at 30°C together with blanks lacking either the β-glucosidase or the substrate, and finally visualized under ultraviolet light at 366 nm. Saliva was also analysed for inhibitory activities on cyanogenesis. Therefore, 50 mg middle leaflets of *L. corniculatus* or MG-20 were crushed in 700 µl of ice-cold 60 mM citric-acid buffer (pH 6) and centrifuged for 10 min at 21.300 g at 4°C. An aliquot (50 µl) of the supernatant was mixed with either 50 µl saliva (165 µg), 50 µl distilled water or 50 µl heat-inactivated saliva (10 min at 95°C). Mixtures were pipetted in a 96-well plate, exposed to Feigl-Anger paper at room temperature as described above and blue colour formation was monitored following exposure for 0, 60 and 120 min.
